# An Observational Cohort Study of the Kynurenine to Tryptophan Ratio in Sepsis: Association with Impaired Immune and Microvascular Function

**DOI:** 10.1371/journal.pone.0021185

**Published:** 2011-06-22

**Authors:** Christabelle J. Darcy, Joshua S. Davis, Tonia Woodberry, Yvette R. McNeil, Dianne P. Stephens, Tsin W. Yeo, Nicholas M. Anstey

**Affiliations:** 1 Global Health Division, Menzies School of Health Research and Charles Darwin University, Darwin, Northern Territory, Australia; 2 Division of Medicine, Royal Darwin Hospital, Darwin, Northern Territory, Australia; 3 Intensive Care Unit, Royal Darwin Hospital, Darwin, Northern Territory, Australia; University of California Los Angeles, United States of America

## Abstract

Both endothelial and immune dysfunction contribute to the high mortality rate in human sepsis, but the underlying mechanisms are unclear. In response to infection, interferon-γ activates indoleamine 2,3-dioxygenase (IDO) which metabolizes the essential amino acid tryptophan to the toxic metabolite kynurenine. IDO can be expressed in endothelial cells, hepatocytes and mononuclear leukocytes, all of which contribute to sepsis pathophysiology. Increased IDO activity (measured by the kynurenine to tryptophan [KT] ratio in plasma) causes T-cell apoptosis, vasodilation and nitric oxide synthase inhibition. We hypothesized that IDO activity in sepsis would be related to plasma interferon-γ, interleukin-10, T cell lymphopenia and impairment of microvascular reactivity, a measure of endothelial nitric oxide bioavailability. In an observational cohort study of 80 sepsis patients (50 severe and 30 non-severe) and 40 hospital controls, we determined the relationship between IDO activity (plasma KT ratio) and selected plasma cytokines, sepsis severity, nitric oxide-dependent microvascular reactivity and lymphocyte subsets in sepsis. Plasma amino acids were measured by high performance liquid chromatography and microvascular reactivity by peripheral arterial tonometry. The plasma KT ratio was increased in sepsis (median 141 [IQR 64–235]) compared to controls (36 [28–52]); p<0.0001), and correlated with plasma interferon-γ and interleukin-10, and inversely with total lymphocyte count, CD8+ and CD4+ T-lymphocytes, systolic blood pressure and microvascular reactivity. In response to treatment of severe sepsis, the median KT ratio decreased from 162 [IQR 100–286] on day 0 to 89 [65–139] by day 7; p = 0.0006) and this decrease in KT ratio correlated with a decrease in the Sequential Organ Failure Assessment score (p<0.0001). IDO-mediated tryptophan catabolism is associated with dysregulated immune responses and impaired microvascular reactivity in sepsis and may link these two fundamental processes in sepsis pathophysiology.

## Introduction

Sepsis is a systemic inflammatory response to infection [1]. Despite advances in its management, severe sepsis still has a mortality rate of 30–50% [2], [3], [4]. Both immune and endothelial dysfunction are thought to contribute to the high mortality rate in sepsis [5], [6], however the underlying mechanisms are not completely understood.

Tryptophan is an essential amino acid that is central to cellular respiration [7] and neurotransmission [8], and is a key immune mediator. During inflammation, tryptophan is metabolised by indoleamine 2,3-dioxygenase (IDO) to the toxic metabolite kynurenine. IDO activity is measured by the ratio of kynurenine to tryptophan (the KT ratio). Endothelial cells, monocytes, renal tubular epithelial cells and hepatocytes express IDO in response to interferon-γ [9], [10], [11], [12], [13] and IL10 stabilises IDO expression [14].

IDO activity regulates a number of immune responses. Increased IDO activity inhibits T cell function [15] and proliferation [14], [16], [17] and contributes to T cell apoptosis [18]. Furthermore, elevated IDO activity inhibits nitric oxide synthase and vice versa [19], [20], [21]. Recent isotope studies have shown that systemic NO production is either reduced or unchanged in human sepsis compared with healthy controls [22], [23], [24].

In addition to regulating the immune response, IDO activity may also regulate endothelial function. Kynurenine, a metabolite of IDO, has recently been described as an endogenous vasorelaxing factor [9]. Increased IDO activity would therefore be expected to directly decrease systemic vascular resistance. Additionally, as IDO inhibits NOS, IDO may indirectly affect endothelial function by impairing NO-dependent microvascular reactivity. NO is essential for normal endothelial function and NO-dependent microvascular reactivity has been previously shown to be impaired in patients with sepsis, in proportion to disease severity [25], [26]. Finally, plasma kynurenine concentrations have been associated with markers of endothelial dysfunction in patients with end-stage renal disease [27].

IDO activity correlates with disease severity in patients with chronic inflammatory diseases such as human immunodeficiency virus [28], systemic lupus erythematosus [29] and malignancy [30], but little is known about IDO activity in acute inflammatory states. A raised KT ratio has recently been reported in patients with bacteremia [31].

We investigated the relationship between the KT ratio and disease severity in sepsis. We hypothesised that the KT ratio would be related to IFN-γ and IL10 concentrations, and inversely related to both T cell lymphopenia and microvascular reactivity, a measure of endothelial NO bioavailability.

## Methods

### Participants

We evaluated patients with sepsis and hospital controls who were part of a previously reported study of endothelial function in sepsis [25]. Sepsis patients had suspected or proven infection and the presence of two or more criteria for the systemic inflammatory response syndrome (SIRS) within the last 4 hours [1]. Severe sepsis patients had organ dysfunction or shock at the time of enrolment according to the American College of Chest Physicians/Society of Critical Care Medicine criteria [1], [32]. Sepsis severity was estimated using the Acute Physiology and Chronic Health Evaluation (APACHE) II score from the first 24 hours of admission and daily modified Sequential Organ Failure Assessment (SOFA) score [33]. Patients were enrolled within 24 hours of ICU admission or within 36 hours of ward admission. Control subjects were recruited from hospital patients who had not met SIRS criteria within the last 30 days and who had no clinical or laboratory evidence of inflammation or infection. Written informed consent was obtained from all participants or next of kin. All sepsis patients had undergone resuscitation and were haemodynamically stable at the time of study enrolment. The study was approved by the Human Research Ethics Committee of Menzies School of Health Research and the Department of Health and Community Services.

### Blood collection and lymphocyte counts

Venous blood was collected in lithium heparin tubes at enrolment, day 2–4, and day 7 until discharge from the hospital or death. Whole blood differential white cell counts were measured by Coulter Counter. Lymphopenia was defined as an absolute lymphocyte count less than 1.2×10^3^/µL [34]. Plasma was separated and stored at −80°C.

Lymphocytes were analysed in more detail in a subset of patients from whom samples were processed within 30 minutes of collection, matched for age and gender. Peripheral blood mononuclear cells were separated using Ficoll-Paque™ Plus (GE Healthcare Biosciences, Uppsala, Sweden) and cryopreserved in fetal calf serum and dimethyl sulfoxide. Cells were thawed and stained with appropriate antibodies and analysed on a FACSCalibur flow cytometer (Becton Dickinson Immunocytometry Systems, MA, USA). Antibodies were sourced from Biolegend, California, USA (CD3, CD16 and CD56) or BD Biosciences Pharmingen, California, USA (CD4 and CD8). Results were analysed using Flow Jo software (Tree Star, Oregon, USA). T cells were defined as CD3+ lymphocytes and natural killer cells were defined as CD3−CD16+CD56+ lymphocytes.

### Tryptophan and kynurenine measurements

Plasma tryptophan and kynurenine concentrations were measured by High Pressure Liquid Chromatography (HPLC; Shimadzu, Kyoto, Japan) with UV (250 nm) and fluorescence (excitation 250 nm, emission 395 nm) detection, using a method modified from van Wandelen and Cohen [35]. The kynurenine to tryptophan (KT) ratio was calculated by dividing the kynurenine concentration (µmol/L) by the tryptophan concentration (µmol/L) and multiplying the quotient by 1000 [28], [36], [37].

### Plasma cytokine measurements

Concentrations of plasma IFN-γ, IL6 and IL10 were determined using a cytometric bead array (Human Th1/Th2 Cytokine Kit II, BD Biosciences Pharmingen, CA, USA) and a FACSCalibur flow cytometer (Becton Dickinson Immunocytometry Systems, MA, USA). Results were analysed using FCAP array version 1.0.1 (Soft Flow Hungary for Becton Dickinson Biosciences). The lower limits of detection (LLD) of the assay were 2.5 pg/mL for IFN-γ and 10 pg/mL for IL6 and IL10. Values below the LLD were assigned a value halfway between zero and the LLD for statistical analysis. Cytokines were only measured if plasma had been frozen within 2 hours of collection.

### Measurement of endothelial function

Sepsis patients underwent serial bedside reactive hyperemia peripheral arterial tonometry (RH-PAT) measurements at enrolment, day 2–4, and day 7 [25]. Control patients had the same assessment at a single time point. RH-PAT (Itamar Medical, Caesarea, Israel) is a non-invasive operator-independent method of assessing endothelial function. Endothelial function is defined by the ability of blood vessels to vasodilate in response to an ischemic stress, which invasive studies have demonstrated to be dependent on endothelial cell NO production [38]. RH-PAT is at least 50% NO-dependent [39]. RH-PAT uses finger probes to measure digital pulse wave amplitude detected by a pressure transducer [40], and has been validated against the more operator-dependent flow-mediated dilatation method [41] and with endothelial function in other vascular beds [42].

### Statistical methods

Predefined groups for analysis were severe sepsis, non-severe sepsis (meaning sepsis without evidence of organ dysfunction or shock at enrolment), and hospital controls. Continuous parametric variables were compared using Student's t-test or ANOVA while continuous non-parametric variables were compared using Mann-Whitney, Kruskal-Wallis or Wilcoxon tests as appropriate. Correlations were examined using Pearson's or Spearman's tests for parametric and non-parametric data respectively. As SOFA score was highly right-skewed and no transformation gave a normal distribution, Kendall's tau coefficient for partial correlation was used for multivariate analysis involving SOFA [43]. Linear mixed-effects models were used to examine longitudinal correlations. A 2-sided p-value of <0.05 was considered significant. Analyses were performed using Stata version 10.0 (Stata Corp TX, USA) and Prism version 5.01 (GraphPad Software, CA, USA).

## Results

### Patients

The study included 50 patients with severe sepsis, 30 with non-severe sepsis and 40 hospital controls. The three groups did not differ significantly in age or gender (**Table 1**). Ninety percent of severe sepsis patients and all non-severe sepsis patients were either orally or enterally fed at the time of enrolment; none were receiving parenteral nutrition.

**Table 1:** Baseline clinical characteristics of participants.

	Severe sepsis	Non-severe sepsis	Controls	p value*
Subjects (n)	50	30	40	
Age†	52 (48–57)	50 (46–55)	48 (44–52)	NS
Male – n (%)	29 (58%)	20 (67%)	27 (68%)	NS
Diabetic – n (%)	16 (32%)	7 (23%)	13 (33%)	NS
Mean Arterial Pressure‡	74 (70–82)n = 50	88 (77–104)n = 30	80 (73–93)n = 37	0.001
Systolic Blood Pressure‡	113 (105–132)n = 49	123 (110–140)n = 24	115 (110–128)n = 37	NS
Diastolic Blood Pressure‡	60 (54–68)n = 49	70 (60–90)n = 24	60 (60–75)n = 37	0.002
APACHE II‡	19 (15–23)	7 (5–12)		<0.0001
SOFA score (day 0)‡	6 (3–9)	1 (0–2)		<0.0001
RH-PAT index†	1.59 (1.45–1.73)n = 45	1.86 (1.67–2.05)n = 26	2.04 (1.91–2.18)n = 36	<0.0001
*Causative Organism – n (%)*				NS
None Cultured	23 (46%)	20 (67%)		
Gram Positive Bacterium	14 (28%)	4 (13%)		
Gram Negative Bacterium	13 (26%)	6 (20%)		
*Nutrition – n (%)*				
Oral feeding	29 (58%)	29 (97%)		
Enteral feeding	16 (32%)	1 (3%)		
Nil By Mouth	5 (10%)			

*For difference between all 3 groups by one way analysis of variance.

†Mean (95% confidence interval).

‡Median (interquartile range).

### IDO activity and sepsis severity

**Figure 1:**
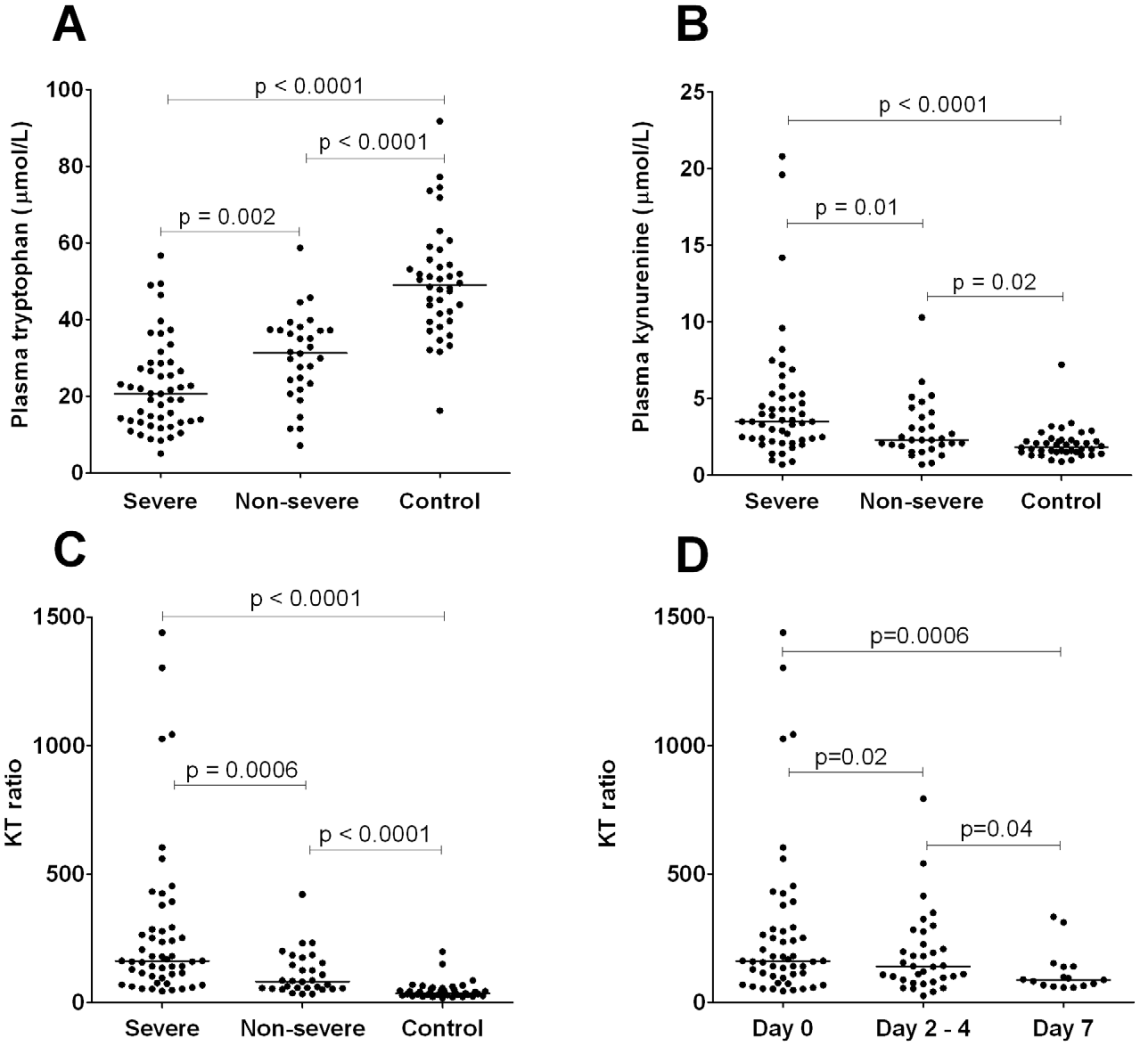
Plasma assessment of tryptophan catabolism. The concentration of plasma tryptophan (Fig. 1A), kynurenine (Fig. 1B) and the KT ratio (Fig. 1C) in 50 severe sepsis patients, 30 non-severe sepsis patients and 40 hospital controls. Fig. 1D shows the KT ratio in severe sepsis patients on admission (n = 50), day 2 (n = 34) and day 7 (n = 16). The KT ratio is determined by dividing the plasma kynurenine concentration (µmol/L) by the plasma tryptophan concentration (µmol/L) and multiplying the quotient by 1000. Horizontal lines represent median values for the group. P value analysis in Figs. 1A–C used a Mann Whitney test, and in Fig. 1D, a paired Wilcoxon test.

Plasma tryptophan concentrations were significantly reduced in patients with sepsis (p<0.0001, **Figure 1** and **Table 2**). In all sepsis patients, plasma tryptophan was inversely related to SOFA score (r = −0.45, p<0.0001). There was no difference in the baseline plasma tryptophan concentrations among severe sepsis patients who were orally fed (n = 29), enterally fed (n = 16) or who were nil by mouth (n = 5).

**Table 2:** Immunological characteristics of participants (median and interquartile range).

	Severe sepsis	Non-severe sepsis	Combined sepsis	Controls	Sepsis vs Control*
n	**50**	**30**	**80**	**40**	
Plasma tryptophan µmol/L	**21** (13–29)	**31** (23–37)	**24** (14–35)	**49** (40–55)	<0.0001
Plasma kynurenine µmol/L	**3.5** (2.4–5.2)	**2.3** (1.9–3.9)	**3.1** (2.1–4.7)	**1.9** (1.5–2.3)	<0.0001
KT ratio	**162** (100–286)	**82** (55–159)	**141** (64–235)	**36** (28–52)	<0.0001
Plasma IFN-γ pg/mL	**8** (1.3–20.1) n = 38	**27** (3–84) n = 29	**9** (3–48) n = 67	**1.3** (1.3–7) n = 37	<0.0001
Plasma IL6 pg/mL	**380** (121–979) n = 38	**136** (44–320) n = 29	**222** (75–596) n = 67	**5** (5-5) n = 37	<0.0001
Plasma IL10 pg/mL	**23** (13–64) n = 38	**5** (5–25) n = 29	**16** (5–41) n = 67	**5** (5 - 5) n = 37	<0.0001
Neutrophils ×10^3^/µL	**13.5** (8.7–20.4) n = 49	**14.1** (9.2–16.3)	**14** (8.8–16.6) n = 79	**5.1** (3.2–6.5) n = 20	0.049
Lymphocytes x ×10^3^/µL	**0.9** (0.5–1.2) n = 49	**1.0** (0.7–1.3)	**0.9** (0.5–1.2) n = 79	**2.1** (1.2–2.2) n = 20	<0.0001
Lymphocyte subsets†	**n = 11**	**n = 12**	**n = 23**	**n = 4**	
T cells ×10^3^/µL	**0.65** (0.34–1.8)	**0.67** (0.34–1.0)	**0.65** (0.34–1.1)	**1.49** (1.0–1.7)	NS
CD4+ T cells ×10^3^/µL	**0.35** (0.17–0.85)	**0.35** (0.17–0.59)	**0.35** (0.18–0.67)	**0.89** (0.52–1.2)	NS
CD8+ T cells ×10^3^/µL	**0.18** (0.07–0.72)	**0.16** (0.10–0.33)	**0.18** (0.1–0.34)	**0.46** (0.31–0.61)	NS
NK cells ×10^3^/µL	**0.07** (0.03–0.12)	**0.06** (0.03–0.17)	**0.06** (0.03–0.11)	**0.08** (0.04–0.20)	NS

*p values, all sepsis vs controls, Mann Whitney test.

†Performed in a subset of patients representative of the entire cohort, as described in methods and results. Severe sepsis n = 11, non-severe sepsis n = 12, control n = 4.

Conversely, plasma kynurenine concentrations were elevated in sepsis patients compared to hospital controls (p<0.0001, **Figure 1** **and** **Table 2**). In all sepsis patients, plasma kynurenine correlated with SOFA score (r = 0.34, p = 0.005). As kynurenine is renally excreted and accumulates in renal failure [44], [45], kynurenine concentrations were tested for relationships with renal impairment. Kynurenine concentrations were significantly higher in patients requiring continuous renal replacement therapy (CRRT) (median 4.5 µmol [IQR 4–5.3]) than in patients not receiving CRRT (2.8 µmol [2.1–4.4]; p = 0.03). In all sepsis patients, kynurenine concentration correlated with plasma creatinine (r = 0.41, p = 0.0002). Nevertheless, the association between plasma kynurenine concentration and SOFA score remained significant even after controlling for creatinine (ktau = 0.24, p<0.01).

IDO activity was significantly increased in sepsis patients (median KT ratio 141 [IQR 64–235]) compared to controls (36 [28–52]) (p<0.0001) and in severe sepsis compared to non-severe sepsis (p = 0.0006, **Table 2**). The baseline KT ratio correlated with APACHE II (r_s_ = 0.51, p<0.0001) and total SOFA scores (r_s_ = 0.54, p<0.0001) in sepsis patients. The KT ratio positively correlated with the hepatic (r_s_ = 0.28, p = 0.01), renal (r_s_ = 0.53, p<0.0001), cardiovascular (r_s_ = 0.42, p<0.0001) and respiratory (r_s_ = 0.36, p = 0.0009) components of the SOFA score but not the coagulation component (r_s_ = 0.13, p = ns).

Of the 80 sepsis patients, 6 died by day 28 of the study. The baseline KT ratio in patients who died (median 270 [IQR 102–431] was not statistically significantly different to those who survived (138 [63–232]; p = 0.2).

In longitudinal analysis of severe sepsis, the KT ratio significantly decreased between day 0 (median 162 [IQR 100–286]) and day 7 (89 [65–139]), p = 0.0006); **Figure 1D**. Among all sepsis patients, decrease in KT ratio correlated with decrease in SOFA score over time (p<0.0001).

### IDO activity and plasma cytokines

Plasma IFN-γ, IL6 and IL10 were all significantly increased in patients with sepsis (**Table 2**). Plasma concentrations of IL1, IL2, IL4 and tumour necrosis factor-α were not significantly increased in this cohort and were not analysed further. Both IL6 and IL10 positively correlated with SOFA score (r_s_ = 0.55, p<0.0001 and r_s_ = 0.55, p<0.0001 respectively) but there was no association between IFN-γ and SOFA score.

In sepsis patients, the KT ratio correlated with plasma IFN-γ (r_s_ = 0.44, p = 0.0002), IL6 (r_s_ = 0.49, p<0.0001) and IL10 (r_s_ = 0.62, p<0.0001). The associations between KT ratio and IL6 and IL10 remained significant after controlling for SOFA score (ktau = 0.30, p<0.003 and ktau = 0.45, p<0.0001 respectively).

In a univariate mixed effects model, the decrease in KT ratio over time correlated with the decrease in IL6 (p<0.0001) and IL10 (p<0.0001) between day 0 and day 7. In a multivariate model, these relationships remained significant after controlling for change in SOFA score (IL6 p = 0.009; IL10 p = 0.02).

### IDO activity and lymphocyte counts

Sepsis patients had increased white blood cell counts (p<0.0001) primarily due to increased circulating neutrophils (p<0.05; **Table 2**), which proliferate in response to bacterial infections [46]. Conversely, sepsis patients had significantly lower total lymphocyte counts compared with hospital controls (p<0.0001, **Table 2**). In all sepsis patients the baseline KT ratio was weakly associated with absolute lymphocyte count (r_p_ = 0.26, p = 0.02). In a linear mixed effects model, absolute lymphocyte count increased as the KT ratio decreased over time (p = 0.001). This relationship persisted after controlling for SOFA score (p = 0.008). When all subjects were grouped according to lymphopenia, lymphopenic patients (n = 63) had a median KT ratio of 128 [IQR 63–236], compared with 59 [33–86] in non-lymphopenic patients (n = 57) (p<0.0001).

As IDO activity contributes to T cell apoptosis [18], we examined the relationship between KT ratio and lymphocyte subsets. Peripheral blood mononuclear cells were analysed from 23 of the 80 sepsis patients whose blood had been processed within 30 minutes of collection. This subset of patients was representative of the cohort in terms of age, gender distribution, total lymphocyte count and KT ratio. In this subset of patients, the KT ratio negatively correlated with absolute numbers of lymphocytes (r_p_ = −0.54, p = 0.007), T cells (r_p_ = −0.53, p = 0.01), CD4+ T cells (r_p_ = −0.50, p = 0.01), CD8+ T cells (r_p_ = −0.49, p = 0.02) and natural killer cells (r_p_ = −0.46, p = 0.03) (**Table 2**).

### IDO activity and endothelial function

In sepsis, the KT ratio at baseline correlated inversely with NO-dependent microvascular reactivity (r_s_ = −0.45, p = 0.001) even after controlling for disease severity (using SOFA score; p = 0.001). In a multivariate mixed effects model controlling for SOFA score, improvement in KT ratio between day 0 and day 7 correlated with improvement in microvascular reactivity (p = 0.001). In all sepsis patients, there was an inverse association between the baseline KT ratio and mean arterial pressure (r_s_ = −0.29, p = 0.009) and diastolic blood pressure (r_s_ = −0.29, p = 0.01) but no association with systolic blood pressure.

## Discussion

**Figure 2:**
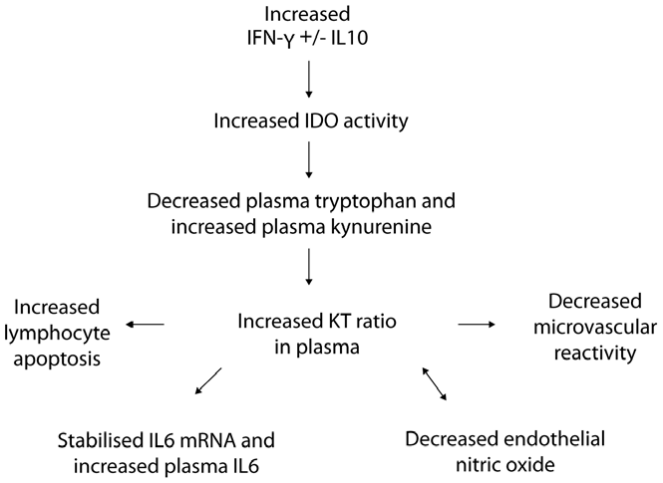
Proposed model of tryptophan catabolism in sepsis. IDO = Indoleamine 2,3-dioxygenase, IL6 = interleukin-6, IL10 = interleukin-10, IFN-γ = interferon gamma and NO = nitric oxide.

IDO activity is increased in sepsis, in proportion to disease severity. IDO-mediated tryptophan catabolism is associated with dysregulated immune responses and impaired microvascular reactivity in sepsis. IFN-γ and IL10 are associated with, and may contribute to, increased IDO activity in sepsis. The independent inverse longitudinal association with total lymphocyte counts suggests a potential role in sepsis-associated lymphopenia. Similarly, the independent inverse association between the KT ratio and microvascular reactivity suggests that IDO activity may also contribute to impaired endothelial function in sepsis. Based on these associations we propose a model of interpretation outlined in **Figure 2**.

Increased expression of IFN-γ [47], IL6 [48], [49] and IL10 [14] have each been associated with increased tryptophan catabolism by IDO in other disease states. In sepsis patients in our study, IFN-γ concentration correlated with the KT ratio only at baseline, whereas IL6 and IL10 correlated with the KT ratio both at baseline and longitudinally. Our findings agree with the *in vitro* literature, where IFN-γ induces IDO [10], [47]. Although under certain conditions, IL-10 has been reported to suppress IDO activity [50], our findings support the majority of in vitro studies which have shown that IL-10 induces or stabilises IDO [14], [51], [52], [53]. The high IFN-γ associated with early sepsis [54] may lead to increased IDO activity while high IL10 may sustain or potentially enhance IDO activity [53] throughout the course of the disease. The role of IL6 in IDO expression is unclear. Orabona *et al.* suggest that IL6 inhibits IDO activity by increasing murine dendritic cell SOCS3 expression, which drives IDO breakdown [55]. On the other hand, a low tryptophan environment created by IDO activity stabilises IL6 mRNA and increases IL6 responses [56]. Given the conflicting evidence in these and other studies regarding IL6 and IDO, we investigated the relationship between the KT ratio and IL6 in sepsis patients. The strong positive correlation between plasma KT ratio and IL6 concentration is consistent with findings in murine models of sepsis where IDO−/− mice or mice treated with IDO inhibitors have lower plasma IL6 concentrations [57], [58].

We report that the high KT ratio in sepsis is associated with a decreased lymphocyte count, independent of disease severity, a finding similar to that found in patients with trauma [37], human immunodeficiency virus [28] and cancer [59]. Previous studies in sepsis have associated lymphopenia with disease severity [60], duration of ICU stay [60] and mortality [61] and prevention of lymphocyte apoptosis improves survival in animal models of sepsis [62], [63], [64], [65]. T cells co-cultured with IDO-producing cells have reduced proliferation and increased death [66], [67]. Both high kynurenine concentrations and low tryptophan concentrations appear to contribute to T cell death. *In vivo*, kynurenine treatment in mice depletes overall thymocyte counts and, *in vitro*, thymocytes die of apoptosis when cultured in media with kynurenines [18]. Furthermore, T cells cultured in low tryptophan media have reduced proliferation and increased apoptosis via activated GCN2 kinase [68], [69]. These *in vitro* studies suggest a potential mechanism through which increased IDO activity may contribute to lymphopenia and its deleterious consequences in sepsis.

IDO activity regulates vascular tone in sepsis. In this study IDO activity in sepsis patients correlated with diastolic blood pressure but not systolic blood pressure. This is consistent with the recent finding that kynurenine is a vascular relaxation factor [9]. Another important regulator of endothelial function in sepsis is NO. There is significant cross-talk between IDO and NOS, with IDO activity inhibiting both expression and activity of NOS [19], [20], [21] and vice versa. We found the KT ratio in sepsis is inversely associated with microvascular reactivity as measured by RH-PAT, which is at least 50% dependent on endothelial NO production [70]. Increased IDO activity in sepsis may regulate vascular tone directly, via the vasorelaxing effects of kynurenine, and indirectly, by impairing NO-dependent microvascular reactivity. Increased plasma kynurenine concentrations may further impede endothelial function in sepsis by mediating adhesion of monocytes and neutrophils to the vascular endothelium [71].

A limitation of this study is that we did not directly measure IDO expression. However, the KT ratio is an established measure of systemic IDO activity [28], [72] with tissue IDO expression and activity directly correlated with plasma KT ratio in multiple human disease states, including celiac disease [73], hepatitis C [11] and pre-eclampsia [74]. There are several possible sources of IDO activity in sepsis patients including the endothelium, kidney, liver, lungs and leukocytes [9], [10], [11], [12], [13], [53], although a recent study was unable to detect spontaneous IDO expression in PBMC from sepsis patients [75]. Importantly, the effects of the high KT ratio in sepsis on immune function and endothelial function would be the same whether the high KT ratio was the result of increased IDO activity alone or in combination with decreased feeding and impaired renal excretion of kynurenine. Furthermore, it is unlikely that nutritional deficiency and renal impairment accounted for the differences we found, because controlling for these factors made no difference to our results.

In our study the KT ratio was not significantly associated with mortality. Consistent with the previously reported low mortality from severe sepsis in our ICU [32], [76], there were few deaths in our study. This suggests that our study was under-powered to examine the relationship between IDO activity and mortality. However, in a study with higher numbers of deaths, Hattunen and colleagues found a clear association between plasma KT ratio and risk of death in sepsis [31].

The generation of a low tryptophan environment may be a maladaptive host response to infection. While growth of some bacterial species is inhibited by low tryptophan [77], most can synthesize tryptophan [78] and others have specialized tryptophan transport systems [79]. In murine models of sepsis, IDO−/− mice have significantly increased survival compared to wild type mice [58] and treatment of wild-type mice with IDO inhibitors such as 1-methyl-tryptophan [58] or ethyl pyruvate also significantly increase survival [57]. The KT ratio is significantly higher in bacteremic patients with a fatal outcome [31] and we and others have demonstrated that the KT ratio is associated with disease severity in sepsis [31], [75], [80]. Together, this evidence supports the hypothesis that increased IDO activity is a deleterious host response in human sepsis. IDO inhibitors are being considered as potential adjunctive cancer treatments [81] and these treatments may also have therapeutic potential in sepsis.

### Conclusion

IDO activity is elevated in sepsis and associated with disease severity, T cell lymphopenia and microvascular dysfunction. Because excessive IDO activity is associated with both immune and endothelial dysfunction, increased tryptophan catabolism may link these two key aspects of sepsis pathophysiology. Modulation of IDO activity warrants investigation as a therapeutic strategy in sepsis.
